# Antioxidant Activity and Proanthocyanidin Profile of *Selliguea feei* Rhizomes

**DOI:** 10.3390/molecules18044282

**Published:** 2013-04-11

**Authors:** Caili Fu, Hongyu Wang, Wei Ling Ng, Lixia Song, Dejian Huang

**Affiliations:** 1 College of Bioscience & Biotechnology, Fuzhou University, Fuzhou 350108, Fujian, China; 2 Department of Chemistry, National University of Singapore, 3 Science Dr. 3, Singapore 117543, Singapore

**Keywords:** *Selliguea feei*, proanthocyanidins, thiolysis, antioxidant activity

## Abstract

Proanthocyanidins from the rhizomes of *Selliguea feei* (PSFs) were solvent-extracted and fractionated by Sephadex LH-20 column chromatography to give a 2.42% isolated yield (dry matter basis). ^1^H-NMR spectroscopy revealed the mean degree of polymerization (mDP) to be 2.6. ^13^C-NMR analysis showed typical signals for afzelechin/epiafzelechin units. Clear peaks at 76 ppm and 84 ppm indicated that both stereoisomers (afzelechin/epiafzelechin) are present. In agreement with the NMR spectra, the ESI-MS spectrum indicated that PSFs are mainly monomers to trimers consisting of afzelechin/epiafzelechin units with A-type and B-type interflavanyl linkages. A trimer was purified and identified as demethylated selligueain B. Thiolysis confirmed the structure and the thiolytic products, methyl 2-[(2*R*, 3*R*, 4*S*)-3,5,7-trihydroxy-2-(4-hydroxyphenyl)chroman-4-yl]acetate (**1**) and 4β-(carboxymethyl)sulphanylepiafzelechin-(2β→O→7,4β→8)-epiafzelechin methyl ester (**2**), were purified and characterized. Selligueain A, demethylated selligueain B, compounds **1** and **2** possess high antioxidant capacity at 1.18 × 10^4^, 1.16 × 10^4^, 0.95 × 10^4^ and 1.29 × 10^4^ µmol TE/g, respectively.

## 1. Introduction

**Figure 1:**
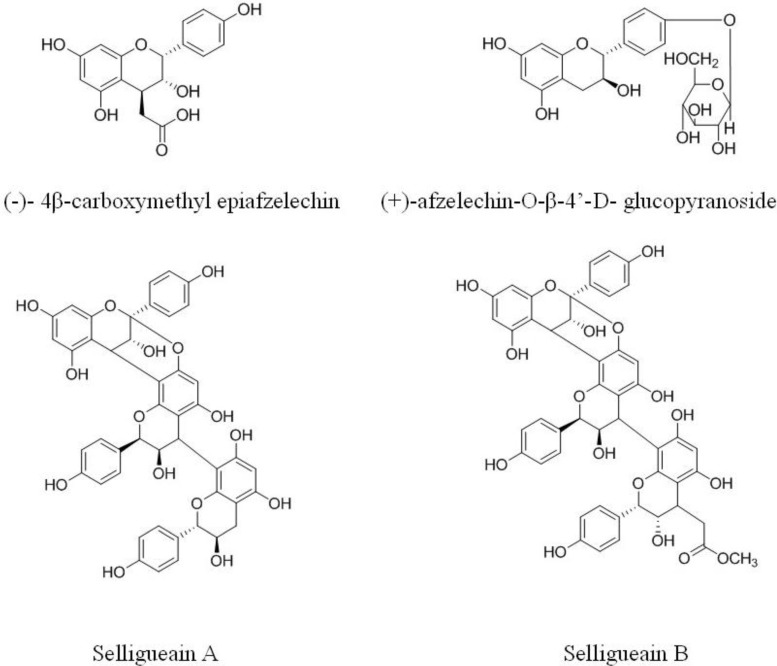
Flavanol monomers and proanthocyanidins reported in *Selliguea feei*.

*Selliguea feei* is a species of edible fern belonging to the Polypodiaceae family. It is a perennial plant that inhabits the forests of Indonesia and Philippines. In western Java, tea made from the rhizomes of *S. feei* is traditionally used as a male tonic. An A-type propelargonidin trimer, selligueain A (Figure 1) was isolated from the rhizomes of *S. feei* and shown to be nontoxic in preliminary acute toxicity tests in mice [1]. In addition, it was reported that selligueain A possesses anti-inflammatory and analgesic activities [2]. Besides selligueain A, other four compounds, selligueain B, (−)-4β-carboxymethyl epiafzelechin, (+)-afzelechin-O-β-4'-d-glucopyranoside (Figure 1) and kaempferol-3-O-β-d-glucopyranoside-7-O-α-L-rhamnopyranoside, were also isolated from this plant [3]. However, the proanthocyanidin profile of the rhizomes of *S. feei* (PSFs) is not clear.

Proanthocyanidins are a class of oligomeric or polymeric flavan-3-ol units found in many plants and foods. They are known to possess numerous bioactivities, including antioxidant, antimicrobial, hypolipidemic and cardioprotective properties [4,5,6,7,8]. We have shown that mangosteen proanthocyanidins and a novel thio-containing conjugate derived from this polymeric material are powerful antioxidants [9,10]. Additionally, several thiolytic products of proanthocyanidins were revealed to be potential candidates for safe neuroprotective agents [11,12]. It is very interesting that the nonphenolic part of the molecule enhanced the capability to penetrate biological membranes and the layers of the skin [12,13]. Reported herein are our results on the PSFs profile and the peroxyl radical scavenging capacity of the various identified components. The thiolytic products were also purified and their antioxidant activities were tested in order to explore some promising applications of these compounds.

## 2. Results and Discussion

Typical solvent extraction and fractionation on Sephadex LH- 20 gave 3.93 g of proanthocyanidin mixture from 200 g of fresh rhizomes of *S. feei*, corresponding to a 1.96% yield based on fresh weight. The proanthocyanidins content is reasonable compared to other food products, except for cocoa and grape seed, whose proanthocyanidin contents are unusually rather high (more than 10% of dry weight) [14,15]. 

**Figure 2:**
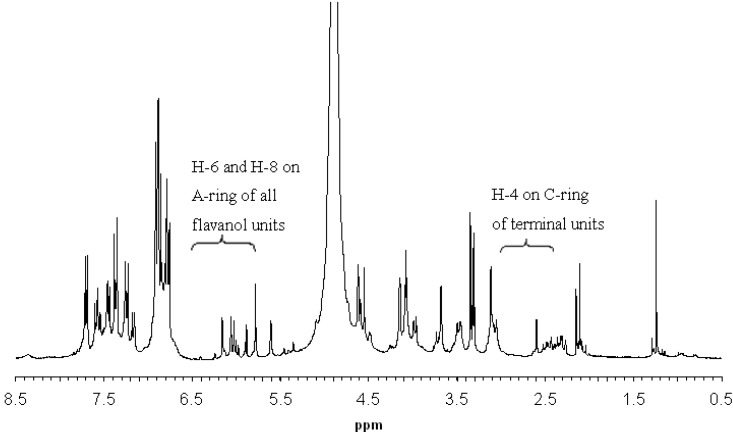
Room temperature 300 MHz ^1^H-NMR spectrum of proanthocyanidins from the rhizomes of *Selliguea feei* (solvent, CD_3_OD).

Proanthocyanidins from the rhizomes of *S. feei* (PSFs) were revealed to be oligomeric proanthocyanidins (OPCs) according to the ^1^H-NMR spectrum in CD_3_OD (Figure 2). The mean degree of polymerization (mDP) of the *S. feei* OPCs was calculated to be 2.6 by integrating the A-ring proton signals between 5.8 and 6.5 ppm and comparing them to the intensity of the H4 signals of the terminal units between 2.4 and 3.0 ppm based on the following equation [16]:

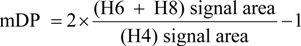
(1)

**Figure 3:**
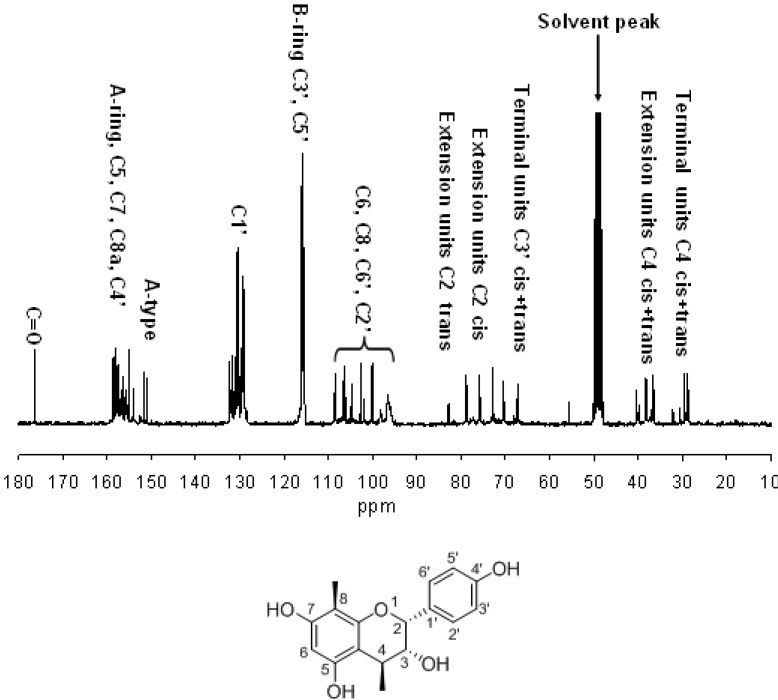
Room temperature 75 MHz ^13^C-NMR spectrum of proanthocyanidins from the rhizomes of *Selliguea feei* (solvent CD_3_OD).

The ^13^C-NMR spectrum (Figure 3) of the PSFs in CD_3_OD shows characteristic propelargonidin peaks. PSFs are mostly composed of propelargonidin (afzelechin/epiafzelechin) units in light of the absence of a clear signal at 145 ppm and 146 ppm, where procyanidin units and prodelphinidin units generally show a typical resonance, respectively [17]. The structural diversity of the linkages (A and B type) and stereochemistry is apparent from the spectrum. Specifically, the C5, C7, C8a and C4'carbons of propelargonidin appear at 160 to 150 ppm. The cluster of peaks around 131 ppm belongs to C1’ and the peak at 116 ppm is assigned to C3' and C5'. The peaks between 110 and 90 ppm is assigned to C8, C6, C6', and C2'. The region between 70 and 90 ppm is sensitive to the stereochemistry of the C ring. The ratio of the 2,3-*cis* to 2,3-*trans* isomers could be determined through the distinct differences in their respective C2 chemical shifts, since C2 gives a resonance line at 76 ppm for the *cis* and at 84 ppm for the *trans* form. The latter is clearly visible in the spectrum, indicating that both stereoisomers (afzelechin/epiafzelechin) are present here. The C3s in terminal units generally have their chemical shift around 67 ppm. The C4 atoms of the extension units appeared at 37 ppm, while the terminal C4 exhibits multiple lines around 29 ppm [17]. A typical B-type interflavanyl linkage was indicated from shift of the C2 at 78 ppm and an obvious A-type linkage was indicated from the signals at 151–152 ppm due to C5 and C7 of the A ring involved in the double linkage. The chemical shift of the ketal carbon (C2) formed as a result of this additional bond observed at 104.7 ppm provided further support for an A-type linkage.

**Figure 4:**
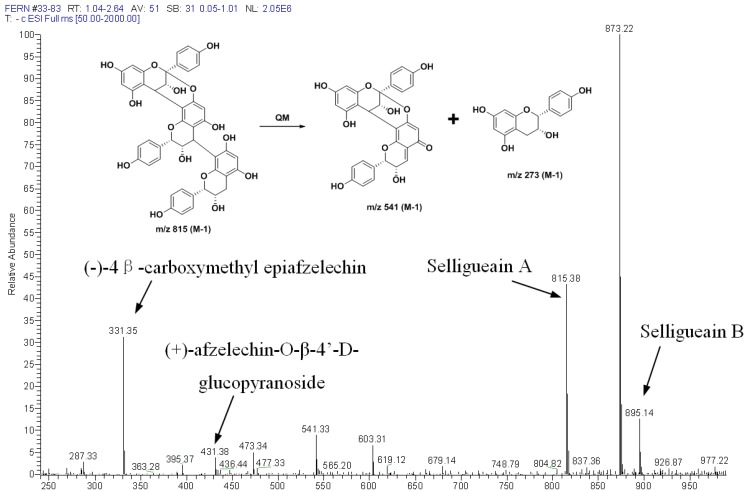
ESI/MS spectra of proanthocyanidins from the rhizomes of *Selliguea feei* recorded in the negative ion mode and the possible fragmentation pathway for selligueain A.

In order to determine the FSPs profile, further characterization was performed by means of ESI/MS and MALDI-TOF MS spectrometry. Analysis was performed in the negative ion mode as proanthocyanidin molecules are better detected by this method than in the positive ion mode. Figure 4 shows the ESI-MS spectrum of the FSPs. Consistent with the NMR spectra, abundant ions were observed from *m/z* 331 to 873, corresponding to the molecular masses of propelargonidins with DP 1–3. The peak at *m/z* = 815 is due to selligueain A (trimer) while the peak at *m/z* = 889 is from selligueain B. (+)-Afzelechin-O-β-4'-d-glucopyranoside and (−)-4β-carboxymethyl epiafzelechin are present at *m/z* = 473 and *m/z* = 331, respectively. The peak at *m/z* = 603 may be due to A-type carboxymethyl propelargonidin dimer, while *m/z* = 541 can be from fragmentation of selligueain A (*m/z* 815) after quinone-methide (QM) cleavage of the interflavanyl bond, as shown in Figure 4. In addition, there is another proanthocyanidin trimer present, as seen from the peak at *m/z* = 873. It corresponds to the most abundant ion. In fact, higher molecular weight proanthocyanidins are more difficult to detect with a good precision in the same spectra as their singly charged ions are often observed with a weak intensity, so this ion should correspond to a proanthocyanidin with high ratio. According to the ^13^C NMR spectra of PSFs, we proposed the structure of this trimer to be demethylated selligueain B, a rare trimer only found previously in another fern [18]. After purification on silica gel, this trimer exhibits spectral (UV, IR, ^1^H-NMR, ^13^C-NMR, ESI-MS) data comparable to published values [18]. There are almost no proanthocyanidin signals after *m/z* 900. MALDI-TOF MS results also indicated that there is no obvious signal corresponding to higher molecule weight polymers, which agrees with the ^1^H- and ^13^C-NMR spectra. 

Thiolysis of the demethylated selligueain B with methyl thioglycolate produced a novel monomer **1** and thioether **2**. The molecular weight of the thioether **2** is determined from ESI-MS spectroscopy as 647 from the anionic mode MS. This thiolytic product is still an A-type propelargonidin dimer since only the B-type interflavanyl bond is readily cleaved under these acidic conditions [19,20]. When the thiolysis was carried out in methanol the desired terminal unit was not obtained. Instead, the methyl ester **1** was isolated as a new compound. Apparently under the acidic methanolic conditions, the depolymerization and esterification occurred in one pot to afford to observed product. The unesterified product can be generated via depolymerization in none-alcoholic media such as 1,4-dioxane. 

**Figure 5:**
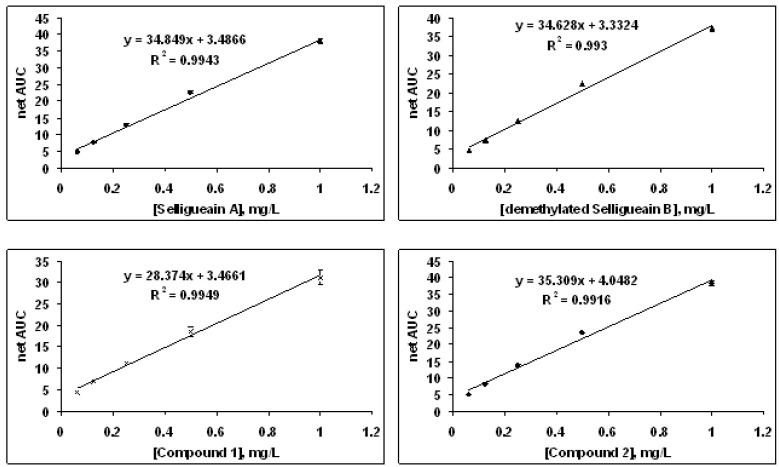
Linear relationships of compounds from *Selliguea feei versus* area under the curve.

The peroxyl radical scavenging capacity of selligueain A, the demethylated selligueain B and the thiolytic products was determined using an oxygen radical absorbance capacity (ORAC) assay in order to investigate the mechanism. The kinetic curves from the ORAC assay show a dose dependent response with a clear lag phase compared to a Trolox standard. The net area under the curve has an excellent linear relationship with the concentration of proanthocyanidins and the thiolytic products (Figure 5). Selligueain A and the demethylated selligueain B, the novel monomer **1** and propelargonidin dimer **2** are potent peroxyl radical scavengers as evidenced by the high oxygen radical scavenging capacity values of 1.18 × 10^4^, 1.16 × 10^4^, 0.95 × 10^4^ and 1.29 × 10^4^ µmol TE/g, respectively. The ORAC values of mangosteen oligomeric proanthocyanidins and commercially available grape seed proanthocyanidins were 1.7 × 10^4^, 1.0 × 10^4^ μmol TE/g, respectively [9]. Therefore, these components derived from the rhizomes of *Selliguea feei* may be promising antioxidants.

Proanthocyanidins isolated from different plants show various degree of polymerizations and characteristic monomeric units. Besides the *S. feei* proanthocyanidins, red kidney bean, strawberry and cinnamon are typical foods containing propelargonidin units [19,21,22]. Proanthocyanidins in red kidney bean have been found to contain the highest proportion of (epi)afzelechin (14.6%) among 88 different kinds of foods. Propelargonidins contribute about 8.2–11.6% of the proanthocyanidins in pinto bean, small red bean, and red kidney bean. The principal proanthocyanidins in these foods are, however, procyanidins [19]. Proanthocyanidins only consisting of afzelechin/epiafzelechin units are rare. A highly sweet proanthocyanidin trimer from the rhizomes of *S. feei*, selligueain A, has been reported to be anti-inflammatory and analgesic, which could be used to treat rheumatism [2]. As unusual secondary metabolites, PSFs and their derivatives warrant further investigation regarding their therapeutic potential and use in chronic disease prevention or as food additives.

## 3. Experimental

### 3.1. Instruments

^1^H and ^13^C-NMR spectra were recorded in deuterated methanol for proanthocyanidins and in deuterated acetone for thiolytic products with a Bruker AC300 spectrometer (Karlsruhe, Germany) operating at 300 and 75 MHz, respectively. The electron spray ionization mass spectra (ESI-MS) were obtained from a Finnigan/MAT LCQ ion trap mass spectrometer (San Jose, CA, USA). The heated capillary and voltage were maintained at 250 °C and 4.5 kV, respectively. The full-scan mass spectra from *m/z* 50 to 2000 were recorded. The proanthocyanidins from the rhizomes of *S. feei* (PSFs) were dissolved in methanol and the solution was introduced into the ion spray source with a syringe (100 uL). High resolution mass spectra (HRMS) were obtained on a Finnigan (MAT 95XL-T) high resolution (60,000), 5KV Double Focusing Reversed Nier–Johnson Geometry Mass Spectrometer. 

### 3.2. Reagents

All solvents used were of reagent grade unless otherwise specified. The rhizomes of *S. feei* were purchased from Indonesia and kept at −20 °C before use. Sephadex^TM^ LH-20 was purchased from GE Healthcare Bi-Sciences AB (Uppsala, Sweden). Methyl thioglycolate, 2,2'-azobis(2-methyl-propionamidine) dihydrochloride (AAPH), and Trolox were purchased from Sigma-Aldrich Chemical Company (St. Louis, MO, USA).

### 3.3. Extraction and Purification of Proanthocyanidins from the Rhizomes of *S. feei*

The rhizomes of *S. feei* (200 g) were meshed and Soxhlet defatted with hexane (500 mL × 2). Condensed tannins were subsequently extracted from the residue by a mixture of acetone/water (7:3, 1,000 mL) for 4 h, according to a classical extraction method for proanthocyanidins [9], and then the mixture was filtered. The extraction was repeated two times and the filtrates were pooled. The acetone in the filtrate was evaporated to yield a slurry mass, which was centrifuged at 3,000 g for 15 min. The supernatant was collected and liquid-liquid extracted with dichloromethane (3 × 200 mL) to further remove other lipophilic compounds. The water phase was collected and concentrated to 40 mL. The crude proanthocyanidin fraction (20 mL) was filtered through a 45 micron porosity filter (Minisart) and then loaded on a Sephadex LH-20 column (100 grams of LH-20, equilibrated with MeOH/water (1:1) for 4h). The column was washed with MeOH/water (1:1) until the eluent turned colorless. The adsorbed proanthocyanidins were then eluted with aqueous acetone (70%, 500 mL). The acetone was removed on a rotary evaporator at 40 °C and the resulting residue freeze-dried to give a light brown powder (3.93 g overall yield from the 200 g fresh the rhizomes of *S. feei*). A portion (2.0 g) of PSFs was applied over sequential silica gel columns, eluting with dichloromethane–methanol (3:1) and acetone-dichloromethane-hexane (5:1:1), respectively, to afford demethylated selligueain B (603 mg). This trimer was identified by comparison of its spectral data (UV, IR, ^1^H and ^13^C-NMR) with published data [18]. In order to test the antioxidant activity, selligueain A was also purified following a previously reported method [1]. 

### 3.4. Thiolysis

**Scheme 1:**
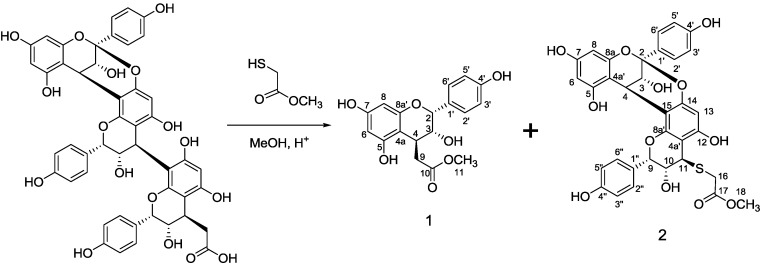
Thiolysis of the demethylated selligueain B.

In a flask (100 mL), the purified demethylated selligueain B (300 mg) was mixed with methanol (30 mL), hydrochloric acid (36%, 0.3 mL), and methyl thioglycolate (0.3 mL). The mixture was heated with stirring at 65 °C for 12 h (Scheme 1). The filtrate was neutralized with 0.1 M NaHCO_3_ to pH 6.5 before it was extracted with ethyl acetate (4 × 50 mL). The combined organic fraction was dried over anhydrous sodium sulphate. Evaporation of the ethyl acetate gave a dark brown residue, which was applied to sequential silica gel columns, eluting with ethyl acetate-hexane (2:1) and dichloromethane–methanol (9:1), respectively, to afford the methyl ester of terminal unit **1** (44 mg) as a light brown solid and a thioether **2** (113 mg) as a light yellow solid.

*Methyl 2-((2R,3R,4S)-3,5,7-trihydroxy-2-(4-hydroxyphenyl)chroman-4-yl)acetate* (**1**). ^1^H-NMR (acetone-d_6_): δ = 7.39 [d, *J* = 8.5 Hz, 2H, C(2', 6')–H], 7.37 [d, *J* = 8.5 Hz, 2H, C(3', 5')–H], 6.04 [d, *J* = 2.3, 1H, C(6)–H], 5.94 [d, *J* = 2.3 Hz, 1H, C(8)–H], 4.96 [s, 1H, C(2)–H], 3.94 [d, *J* = 4.7 Hz, 1H, C(4)–H], 3.74 [d, *J* = 5.7 Hz, 1H, C(3)–H], 3.67 [s, 3H, C(11)–H], 3.06 and 2.52 [two dd, *J*_1_ = 12.5 Hz, *J*_2_ = 3.6 Hz, 2H, C(9)–H]. ^13^C{1H}-NMR (acetone-d_6_): δ = 172.2 (C-10), 157.0 (C-5), 156.9 (C-7), 156.7 (C-8a), 156.0(C-4), 130.3(C-1'), 128.2 (C-2' and C-6'), 114.6 (C-3' and C-5'), 101.5 (C-4a), 95.5 (C-6), 94.9 (C-8), 74.4 (C-2), 69.1 (C-3), 50.7 (C-11), 38.2 (C-4), 35.3 (C-9). MS (ESI, -c): 345 [M−H]^−^. IR (KBr): 3412, 2954, 1715, 1616, 1518, 1468, 1365, 1238, 1172, 1149, 1130, 1050, 1025, 818, 795 cm^−1^. 

*4β-(Carboxymethyl)sulphanyl-epiafzelechin-(2β→O→7,4β→8)-epiafzelechin methyl ester* (**2**). ^1^H-NMR (acetone-d_6_): δ = 7.70 [s, 1H, (C2')-H], 7.67 [s, 1H, (C6')-H], 7.57 [s, 1H, (C2'')-H], 7.54 [s, 1H, (C6'')-H], 6.94 [s, 1H, (C3')-H], 6.91 [s, 1H, (C3')-H], 6.89 [s, 1H, (C3'')-H], 6.86 [s, 1H, (C5'')-H], 6.17 [s, 1H, (C13)-H], 6.09 [d, *J* = 2.3, 1H, (C-6)-H], 6.99 [d, *J* = 2.3, 1H, (C-8)-H], 5.36 [s, 1H, C(9)-H], 4.37 [d, *J* = 3.5, 1H, (C-4)-H], 4.29 [s, 1H, (C-10)-H], 4.17 [s, 1H, (C-3)-H], 4.14 [s, 1H, (C-11)-H]; 3.74 [m, 4H, (C-18, C-19)-H]. ^13^C{^1^H}-NMR (acetone-d_6_): d = 173.4 (C-16), 159.2 (C-4'), 158.6 (C-4''), 157.9 (C-12), 157.6 (C-7), 154.6 (C-5), 154.7 (C-14), 152.3 (C-8a, C-8a'), 132.1 (C-1'', C-1’), 131.3 (C-2'', C-6''), 130.2 (C-2'), 130.0 (C-6'), 116.5 (C-3'', 5''), 115.9 (C-3',5'), 107.7 (C-8), 104.3 (C-4a), 102.0 (C-4a’), 100.7 (C-2), 98.8 (C-6), 98.2 (C-13), 96.9 (C-8), 78.1 (C-9), 71.1 (C-10), 68.0 (C-3), 53.7 (C-18), 45.1 (C-11), 34.9 (C-16), 27.8 (C-4). MS (ESI, -c): 647 [M−H]^−^. IR (KBr): 3391, 2956, 1715, 1615, 1518, 1474, 1449, 1378, 1307, 1230, 1174, 1143, 1120, 1009, 966, 899, 880, 835, 781, 747 cm^−1^.

### 3.5. Antioxidant Capacity Analysis

The oxygen radical absorbance capacity (ORAC) is the excellent method combining both degree of inhibition and inhibition time into a single quantity [23]. Oxygen radical absorbance capacity (ORAC_FL_) assays were carried out on a Synergy HT fluorescent microplate reader with an excitation wavelength of 485 nm and an emission wavelength of 525 nm (Bio-tek Instruments Inc., Winooski, VT, USA). The temperature of the incubator was set at 37 °C. The procedures were based on the modified ORAC_FL_ method [23]. All assays were carried out in triplicate and the data are expressed as micromoles of Trolox equivalents per gram (μmol TE/g). 

## 4. Conclusions

In summary, we have shown that the rhizomes of *Selliguea feei* are a good source of oligomeric proanthocyanidins consisting of afzelechin/epiafzelechin units with A-type and B-type interflavanyl linkages. Two novel thiolytic products and two propelargonidin trimers from the rhizomes of *Selliguea feei* are potent peroxyl radical scavengers as evidenced by the high antioxidant capacity. The chemistry behind these compounds may warrant further study for exploring potential therapeutic applications or as food additives. 
